# Supplementary material for: An appropriate DNA input for bisulfite conversion reveals LINE-1 and Alu hypermethylation in tissues and circulating cell-free DNA from cancers
Source: PLoS One. 2024 Dec 30;19(12):e0316394. doi: 10.1371/journal.pone.0316394 (PMC11684646; doi:10.1371/journal.pone.0316394)
Supplement: S1 Table — The methylation-dependent-specific PCR (MSP) primers (Me-) are designed from the consensus sequences of LINE-1 and Alu (S1 Fig). All non-CpG cytosines were replaced by ‘t” in the forward primers and by “a” in the reverse ones. (PDF) [file pone.0316394.s001.pdf]

S1 Table: An appropriate DNA input for bisulfite conversion reveals *LINE-1* and *Alu* hypermethylation in tissues and circulating cell-free DNA from cancers

Trang Thi Quynh Tran[1,2], Tung The Pham[1], Than Thi Nguyen[1,4], Trang Hien Do[1], Phuong Thi Thu Luu[1], Uyen Quynh Nguyen[2], Linh Dieu Vuong[3], Quang Ngoc Nguyen[3], Son Van Ho[4], Hang Viet Dao[5], Tong Van Hoang[6], Lan Thi Thuong Vo[1,2*]

1 Faculty of Biology, VNU University of Science, Vietnam National University, Hanoi. 2 VNU Institute of Microbiology and Biotechnology. 3 Pathology and Molecular Biology Center, Vietnam National Cancer Hospital. 4 Department of Chemistry, 175 Hospital, Ho Chi Minh City. 5 Endoscopic Centre, Hanoi Medical University Hospital. 6 Institute of Biomedicine and Pharmacy, Ha Dong, Vietnam.

S1 Table. Primer sets and quantitative real-time PCR conditions for quantification of copy number and measurement of methylation level of *LINE-1* and *Alu*. The methylation-dependent-specific PCR (MSP) primers (Me-) are designed from the consensus sequences of *LINE-1* and *Alu* (S1 Fig.). All non-CpG cytosines were replaced by ‘t” in the forward primers and by “a” in the reverse ones

	**Primers**	**Sequence (5’–3’)**	**Amplicon size (bp)**	**PCR conditions**
Quantitative measurement of methylation (MSP primer sets)	Me-Alu-F	CGGtTTAAGAAACGGCGtAttAC	82	95°C 2 min, 40 cycles of (95°C 15 sec, 59C 60 sec)
	Me-Alu -R	aCAATCAaCGAaATTCCGTaaaCG		
	Me-L1-F	AGtAtTTTGGGAGGtCGAGGC	72	
	Me-L1-R	CACCGTaTTAaCCAaaATaaTCTCG		
Quantitative measurement of *LINE-1* copy number	L1-native-F	CTAGCACAGCAGTCTGAGATC	88	95°C 2 min, 40 cycles of (95°C 15 sec, 61°C 30 sec)
	L1-native-R	GTTTACCTAAGCAAGCCTGGG		
Quantitative measurement of cfDI	shortAlu-F	AGGTGAAACCCCGTCTCTAC	78	95°C 2 min, 40 cycles of (95°C 30 sec, 64°C 1 min)
	shortAlu-R	CGAGTAGCTGGGACTACAGG		
	longAlu-F	ACGCCTGTAATCCCAGCACT	205	
	longAlu-R	CAAACTCCGCTTCCCGGGT		
