# Supplementary material for: An appropriate DNA input for bisulfite conversion reveals LINE-1 and Alu hypermethylation in tissues and circulating cell-free DNA from cancers
Source: PLoS One. 2024 Dec 30;19(12):e0316394. doi: 10.1371/journal.pone.0316394 (PMC11684646; doi:10.1371/journal.pone.0316394)
Supplement: S2 Table — Association of clinicopathological characteristics of cancer patients with LINE-1 (A) and Alu (B) methylation status in tissues of breast cancer, colon cancer and lung cancer. (PDF) [file pone.0316394.s002.pdf]

S2 Table: An appropriate DNA input for bisulfite conversion reveals *LINE-1* and *Alu* hypermethylation in tissues and circulating cell-free DNA from cancers

Trang Thi Quynh Tran[1,2], Tung The Pham[1], Than Thi Nguyen[1,4], Trang Hien Do[1], Phuong Thi Thu Luu[1], Uyen Quynh Nguyen[2], Linh Dieu Vuong[3], Quang Ngoc Nguyen[3], Son Van Ho[4], Hang Viet Dao[5], Tong Van Hoang[6], Lan Thi Thuong Vo[1,2*]

1 Faculty of Biology, VNU University of Science, Vietnam National University, Hanoi. 2 VNU Institute of Microbiology and Biotechnology. 3 Pathology and Molecular Biology Center, Vietnam National Cancer Hospital. 4 Department of Chemistry, 175 Hospital, Ho Chi Minh City. 5 Endoscopic Centre, Hanoi Medical University Hospital. 6 Institute of Biomedicine and Pharmacy, Ha Dong, Vietnam.

S2 Table. Association of clinicopathological characteristics of cancer patients with *LINE-1* (A) and *Alu* (B) methylation status in tissues of breast cancer and colon cancer

**(A)**	**Breast cancer (190)**			**Colorectal cancer (132)**		
	Total	Median	P-value	Total	Median	P-value
**Age**						
<50	67	48.703		16	46.337	
≥50	123	53.338	0.4893[b]	116	37.682	0.1536[b]
**NI**	-			-		
**Gender**						
Male	-			82	37.605	0.5708[b]
Female	190	52.110		50	42.092	
**NI**	-					
**Pathological stage**						
I	33	54.116		-		
II	40	74.142		-		
III	13	73.458	0.0038[a]	-		
IV	-			-		
**NI**	104			132		
**Tumour size (Breast cancer)/Tumour stage (Colorectal cancer)**						
pT1	48	48.811		-		
pT2	68	58.827		-		
pT3	-			51	46.095	
pT4	-		0.0131[a]	19	36.543	0.0075[a]
pT1+ pT2	-			26	28.972	
pT3 + pT4	13	81.286		-		
**NI**	61			36		
**Tumour grade**						
I+II	130	50.549		108	38.030	0.1691[b]
III	22	77.694	<0.0001[b]	11	50.928	
**NI**	38			13		

**Nodal status**						
N0	85	52.324		-		
N1	31	50.638	0.3132[a]	-		
N2 + N3	13	61.890		-		
**NI**	61			132		
**Histologic tumour type**						
IDC	134	52.774		-		
Adenocarcinoma	-		0.1950[b]	129	39.034	0.9078[b]
Other	56	48.827		3	31.901	
**NI**	-			-		

** NI: No-information*
** a: Using the Kruskal-Wallis test*
** b: Using the Mann-Whitney U test*

**(B)**	**Breast cancer (201)**			**Colorectal cancer (133)**		
	Total	Median	P-value	Total	Median	P-value
**Age**						
<50	79	49.655		17	46.802	
≥50	122	47.140	0.3594[b]	116	49.845	0.4154[b]
**NI**	-			-		
**Gender**						
Male	-			82	49.006	0.3639[b]
Female	201	47.963		51	53.071	
**NI**	-			-		
**Pathological stage**						
I	32	74.075		-		
II	35	53.962		-		
III	12	47.963	0.0426[a]	-		
IV	-			-		
**NI**	122			133		
**Tumour size (Breast cancer)/Tumour stage (Colorectal cancer)**						
pT1	48	48.467		-		
pT2	63	44.135		-		
pT3	-			50	52.026	
pT4	-		0.4155[a]	18	44.182	0.4861[a]
pT1+ pT2	-			28	46.952	
pT3 + pT4	13	46.652		-		
**NI**	77			37		
**Tumour grade**						
I+II	137	47.303		112	49.845	0.0535[b]
III	21	51.406	0.7057[b]	9	35.758	
**NI**	43			12		
**Nodal status**						
N0	81	47.303		-		
N1	31	41.466	0.0811[a]	-		
N2 + N3	12	39.359		-		
**NI**	77			133		
**Histologic tumour type**						
IDC	144	47.963		-		
Adenocarcinoma	-		0.4740[b]	130	49.501	0.7251[b]
Other	55	47.468		3	48.063	
**NI**	2			-		

* *NI: No-information*
* *a: Using the Kruskal-Wallis test*
* *b: Using the Mann-Whitney U test*
