# Supplementary material for: An appropriate DNA input for bisulfite conversion reveals LINE-1 and Alu hypermethylation in tissues and circulating cell-free DNA from cancers
Source: PLoS One. 2024 Dec 30;19(12):e0316394. doi: 10.1371/journal.pone.0316394 (PMC11684646; doi:10.1371/journal.pone.0316394)
Supplement: S3 Table — Association of clinicopathological characteristics of lung cancer patients with LINE-1 (A) and Alu (B) methylation status in cfDNA. (PDF) [file pone.0316394.s003.pdf]

S3 Table: An appropriate DNA input for bisulfite conversion reveals *LINE-1* and *Alu* hypermethylation in tissues and circulating cell-free DNA from cancers

Trang Thi Quynh Tran[1,2], Tung The Pham[1], Than Thi Nguyen[1,4], Trang Hien Do[1], Phuong Thi Thu Luu[1], Uyen Quynh Nguyen[2], Linh Dieu Vuong[3], Quang Ngoc Nguyen[3], Son Van Ho[4], Hang Viet Dao[5], Tong Van Hoang[6], Lan Thi Thuong Vo[1,2*]

1 Faculty of Biology, VNU University of Science, Vietnam National University, Hanoi. 2 VNU Institute of Microbiology and Biotechnology. 3 Pathology and Molecular Biology Center, Vietnam National Cancer Hospital. 4 Department of Chemistry, 175 Hospital, Ho Chi Minh City. 5 Endoscopic Centre, Hanoi Medical University Hospital. 6 Institute of Biomedicine and Pharmacy, Ha Dong, Vietnam.

S3 Table. Association of clinicopathological characteristics of lung cancer patients with *LINE-1* (A) and *Alu* (B) methylation status in cfDNA

	**(A)**			**(B)**		
	***LINE-1* methylation (296)**			***Alu* methylation (291)**		
	Total	Median	P-value	Total	Median	P-value
**Age**						
<50	49	51.782		47	58.642	
≥50	247	48.655	0.2976[b]	238	55.729	0.4463[b]
NI	-			6		
**Gender**						
Male	179	50.153		170	56.763	
Female	117	48.633	0.5014[b]	113	55.671	0.6532[b]
NI	-			8		
**Pathological stage**						
III	25	69.467		28	53.864	
IV	170	49.713	0.0185[b]	164	55.913	0.2452[b]
NI	101			99		
**Metastasis**						
M0	25	69.467		28	53.864	
M1	170	49.713	0.0185[b]	164	55.913	0.2452[b]
NI	101			99		
**Cancer subtype (Lung cancer)**						
NSCLC	237	50.253		240	55.057	
SCLC	8	67.372	0.6892[b]	6	66.065	0.5199[b]
NI	51			45		

** NI: No-information*
** a: Using the Kruskal-Wallis test*
** b: Using the Mann-Whitney U test*
