# Supplementary material for: An appropriate DNA input for bisulfite conversion reveals LINE-1 and Alu hypermethylation in tissues and circulating cell-free DNA from cancers
Source: PLoS One. 2024 Dec 30;19(12):e0316394. doi: 10.1371/journal.pone.0316394 (PMC11684646; doi:10.1371/journal.pone.0316394)
Supplement: S1 Fig — (A-C) Alignment of the 5’ end region of L1 elements. The alignments include the old L1PA consensus sequence (A), the young L1Hs consensus representative to L1-Ta-0 (B) and L1-Ta-1 (C). The positions of the primers used in this study are shown with arrows, indicating the primers have been well designed to target all L1Hs elements including the L1 consensus sequence (accession no. X58075). (D-F) Alignment of the Alu sequences of young (AluY), intermediate (AluS) and old (Alu J) individual Alu repeats. Based on these Alu consensus sequences, the primer sets specific to methylated Alu sequences were designed. The positions of the primers used in this study are shown with arrows. (PDF) [file pone.0316394.s004.pdf]

S1 Fig: An appropriate DNA input for bisulfite conversion reveals *LINE-1* and *Alu* hypermethylation in tissues and circulating cell-free DNA from cancers

Trang Thi Quynh Tran[1,2], Tung The Pham[1], Than Thi Nguyen[1,4], Trang Hien Do[1], Phuong Thi Thu Luu[1], Uyen Quynh Nguyen[2], Linh Dieu Vuong[3], Quang Ngoc Nguyen[3], Son Van Ho[4], Hang Viet Dao[5], Tong Van Hoang[6], Lan Thi Thuong Vo[1,2*]

1 Faculty of Biology, VNU University of Science, Vietnam National University, Hanoi. 2 VNU Institute of Microbiology and Biotechnology. 3 Pathology and Molecular Biology Center, Vietnam National Cancer Hospital. 4 Department of Chemistry, 175 Hospital, Ho Chi Minh City. 5 Endoscopic Centre, Hanoi Medical University Hospital. 6 Institute of Biomedicine and Pharmacy, Ha Dong, Vietnam.

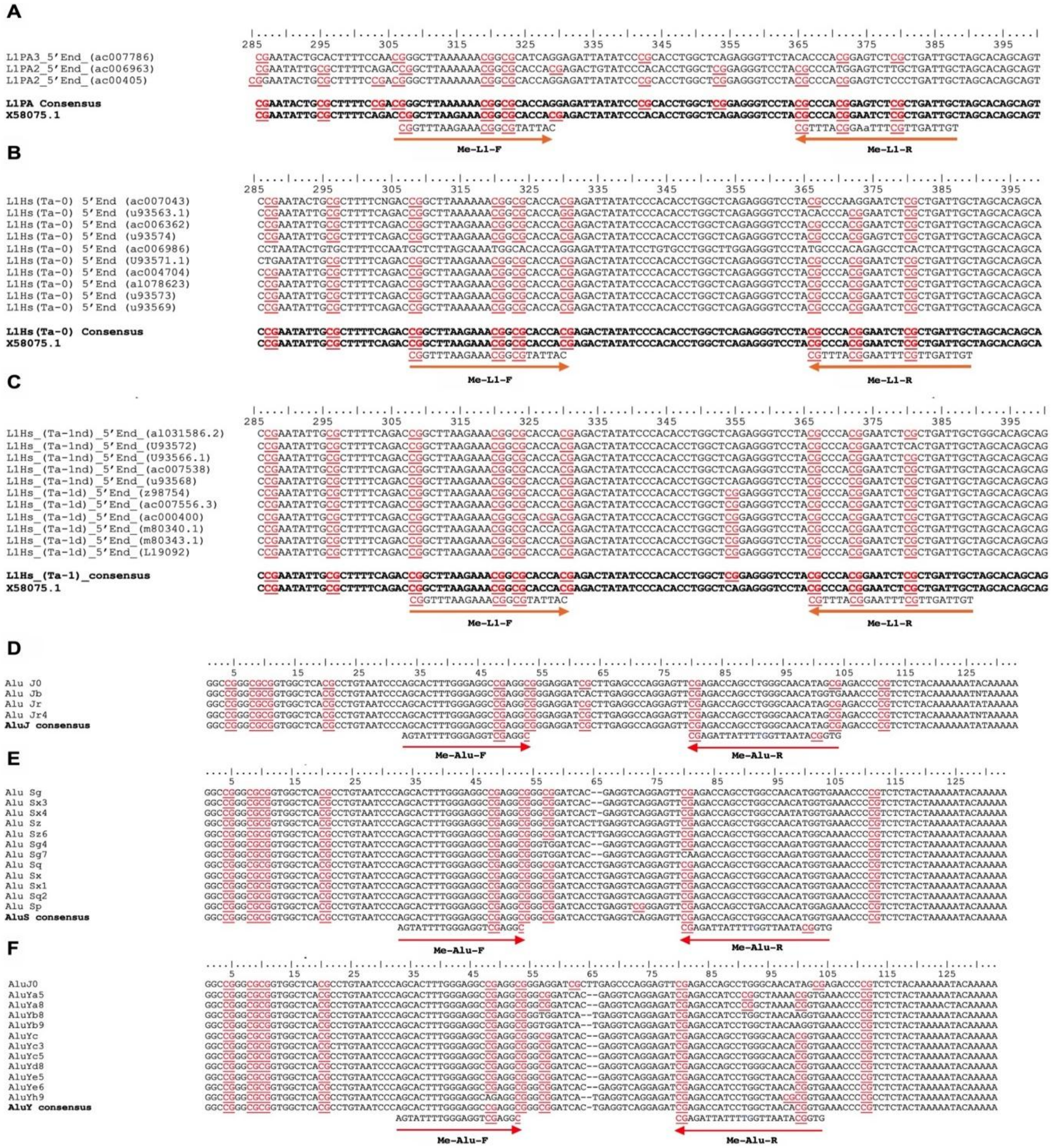

S1 Fig. Identification of *LINE-1* and *Alu* consensus sequences followed by analyses on Repeat Masker [49] and ClustalW. (A-C) Alignment of the 5' end region of *L1* elements. The alignments include the old *L1PA* consensus sequence (A), the young *L1Hs* consensus representative to *L1-Ta-0* (B) and *L1-Ta-1* (C). The positions of the primers used in this study are shown with arrows, indicating the primers have been well designed to target all *L1Hs* elements including the *L1* consensus sequence (accession no. X58075). (D-F) Alignment of the *Alu* sequences of young (*AluY*), intermediate (*AluS*) and old (*Alu J*) individual *Alu* repeats. Based on these *Alu* consensus sequences, the primer sets specific to methylated *Alu* sequences were designed. The positions of the primers used in this study are shown with arrows.
