# Supplementary material for: An appropriate DNA input for bisulfite conversion reveals LINE-1 and Alu hypermethylation in tissues and circulating cell-free DNA from cancers
Source: PLoS One. 2024 Dec 30;19(12):e0316394. doi: 10.1371/journal.pone.0316394 (PMC11684646; doi:10.1371/journal.pone.0316394)
Supplement: S2 Fig — (A) The PCR product was successfully amplified with the primers specific to the native LINE-1 from bisulfite-treated DNA with 5ng, 50ng, and 500 ng, on DNA samples of breast (1), colon (2) and lung tumour (3) tissues. However, no native LINE-1 product was detected when the DNA input was 0.5 ng. (—): Negative control without DNA template. (B). Direct sequencing of PCR products amplified from bisulfite-treated DNA with an input amount of 0.5 ng using the MIP primers and an input amount of 50 ng using the primers specific to the native LINE-1 sequences. Almost non-CpG cytosines remained in DNA input of 50 ng. Rare conversions of non-CpG cytosines were underlined. (PDF) [file pone.0316394.s005.pdf]

S2 Fig: An appropriate DNA input for bisulfite conversion reveals *LINE-1* and *Alu* hypermethylation in tissues and circulating cell-free DNA from cancers

Trang Thi Quynh Tran[1,2], Tung The Pham[1], Than Thi Nguyen[1,4], Trang Hien Do[1], Phuong Thi Thu Luu[1], Uyen Quynh Nguyen[2], Linh Dieu Vuong[3], Quang Ngoc Nguyen[3], Son Van Ho[4], Hang Viet Dao[5], Tong Van Hoang[6], Lan Thi Thuong Vo[1,2*]

1 Faculty of Biology, VNU University of Science, Vietnam National University, Hanoi. 2 VNU Institute of Microbiology and Biotechnology. 3 Pathology and Molecular Biology Center, Vietnam National Cancer Hospital. 4 Department of Chemistry, 175 Hospital, Ho Chi Minh City. 5 Endoscopic Centre, Hanoi Medical University Hospital. 6 Institute of Biomedicine and Pharmacy, Ha Dong, Vietnam.

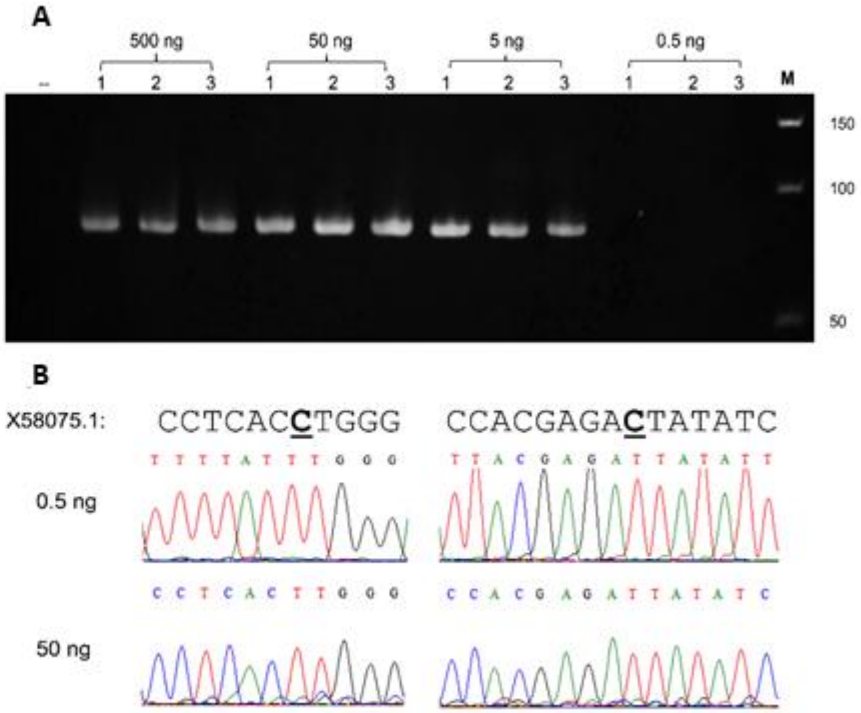

S2 Fig. Detection of the native *LINE-1* sequences after bisulfite conversion. (**A**) The PCR product was successfully amplified with the primers specific to the native *LINE-1* from bisulfite-treated DNA with 5ng, 50ng, and 500 ng, on DNA samples of breast (1), colon (2) and lung tumour (3) tissues. However, no native *LINE-1* product was detected when the DNA input was 0.5 ng. (--): Negative control without DNA template. (**B**). Direct sequencing of PCR products amplified from

bisulfite-treated DNA with an input amount of 0.5 ng using the MIP primers and an input amount of 50 ng using the primers specific to the native *LINE-1* sequences. Almost non-CpG cytosines remained in DNA input of 50 ng. Rare conversions of non-CpG cytosines were underlined.
