# Supplementary material for: An appropriate DNA input for bisulfite conversion reveals LINE-1 and Alu hypermethylation in tissues and circulating cell-free DNA from cancers
Source: PLoS One. 2024 Dec 30;19(12):e0316394. doi: 10.1371/journal.pone.0316394 (PMC11684646; doi:10.1371/journal.pone.0316394)
Supplement: S3 Fig — (A). The PCR product was successfully amplified with the MSP primers specific to the methylated Alu sequences from bisulfite-treated DNA with different DNA quantities of 0.5 ng and 50 ng on DNA samples of breast (1) and colon (2) tumour tissues. (+): Recombinant plasmid containing the methylated Alu sequences used as template. (B). The PCR product was successfully amplified with the primers specific to the native Alu sequences from bisulfite-treated DNA of 50 ng input. However, no native Alu product was detected when the DNA input was 0.5 ng. PC: Positive control with DNA extracted from a blood sample as template. NTC: Negative control without DNA template. (C). Digestion of the PCR products from (B) with the HpaII restriction enzyme, recognizing one CCGG site in the Alu sequences, indicated that some native Alu sequences remained after bisulfite conversion of a DNA input of 50 ng. (PDF) [file pone.0316394.s006.pdf]

S3 Fig: An appropriate DNA input for bisulfite conversion reveals *LINE-1* and *Alu* hypermethylation in tissues and circulating cell-free DNA from cancers

Trang Thi Quynh Tran[1,2], Tung The Pham[1], Than Thi Nguyen[1,4], Trang Hien Do[1], Phuong Thi Thu Luu[1], Uyen Quynh Nguyen[2], Linh Dieu Vuong[3], Quang Ngoc Nguyen[3], Son Van Ho[4], Hang Viet Dao[5], Tong Van Hoang[6], Lan Thi Thuong Vo[1,2*]

1 Faculty of Biology, VNU University of Science, Vietnam National University, Hanoi. 2 VNU Institute of Microbiology and Biotechnology. 3 Pathology and Molecular Biology Center, Vietnam National Cancer Hospital. 4 Department of Chemistry, 175 Hospital, Ho Chi Minh City. 5 Endoscopic Centre, Hanoi Medical University Hospital. 6 Institute of Biomedicine and Pharmacy, Ha Dong, Vietnam.

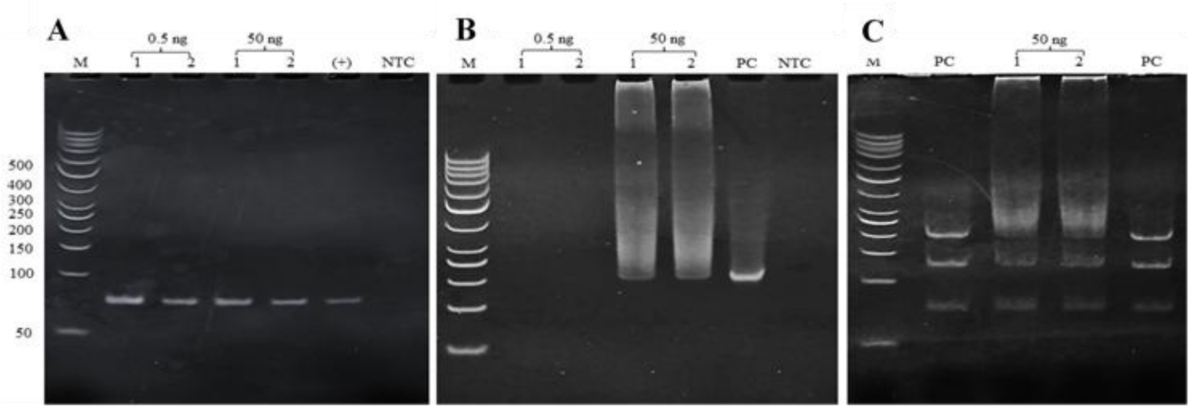

S3 Fig. Detection of the native *Alu* sequences after bisulfite conversion. (**A**). The PCR product was successfully amplified with the MSP primers specific to the methylated *Alu* sequences from bisulfite-treated DNA with different DNA quantities of 0.5 ng and 50 ng on DNA samples of breast (1) and colon (2) tumour tissues. (+): Recombinant plasmid containing the methylated *Alu* sequences used as template. (**B**). The PCR product was successfully amplified with the primers specific to the native *Alu* sequences from bisulfite-treated DNA of 50 ng input. However, no native *Alu* product was detected when the DNA input was 0.5 ng. PC: Positive control with DNA extracted from a blood sample as template. NTC: Negative control without DNA template. (**C**). Digestion of the PCR products from (B) with the *Hpa*II restriction enzyme, recognizing one CCGG site in the *Alu* sequences, indicated that some native *Alu* sequences remained after bisulfite conversion of a DNA input of 50 ng.
