# Supplementary material for: An appropriate DNA input for bisulfite conversion reveals LINE-1 and Alu hypermethylation in tissues and circulating cell-free DNA from cancers
Source: PLoS One. 2024 Dec 30;19(12):e0316394. doi: 10.1371/journal.pone.0316394 (PMC11684646; doi:10.1371/journal.pone.0316394)
Supplement: S4 Fig — LINE-1 (A) and Alu (B) methylation levels in cfDNA do not correlate with ageing in lung cancer patients. Methylation assessments were performed on one microlitre of bisulfite-converted cfDNA. The Spearman’s rank correlation test (A, B) was used in statistical analysis. (PDF) [file pone.0316394.s007.pdf]

S4 Fig: An appropriate DNA input for bisulfite conversion reveals *LINE-1* and *Alu* hypermethylation in tissues and circulating cell-free DNA from cancers

Trang Thi Quynh Tran[1,2], Tung The Pham[1], Than Thi Nguyen[1,4], Trang Hien Do[1], Phuong Thi Thu Luu[1], Uyen Quynh Nguyen[2], Linh Dieu Vuong[3], Quang Ngoc Nguyen[3], Son Van Ho[4], Hang Viet Dao[5], Tong Van Hoang[6], Lan Thi Thuong Vo[1,2*]

1 Faculty of Biology, VNU University of Science, Vietnam National University, Hanoi. 2 VNU Institute of Microbiology and Biotechnology. 3 Pathology and Molecular Biology Center, Vietnam National Cancer Hospital. 4 Department of Chemistry, 175 Hospital, Ho Chi Minh City. 5 Endoscopic Centre, Hanoi Medical University Hospital. 6 Institute of Biomedicine and Pharmacy, Ha Dong, Vietnam.

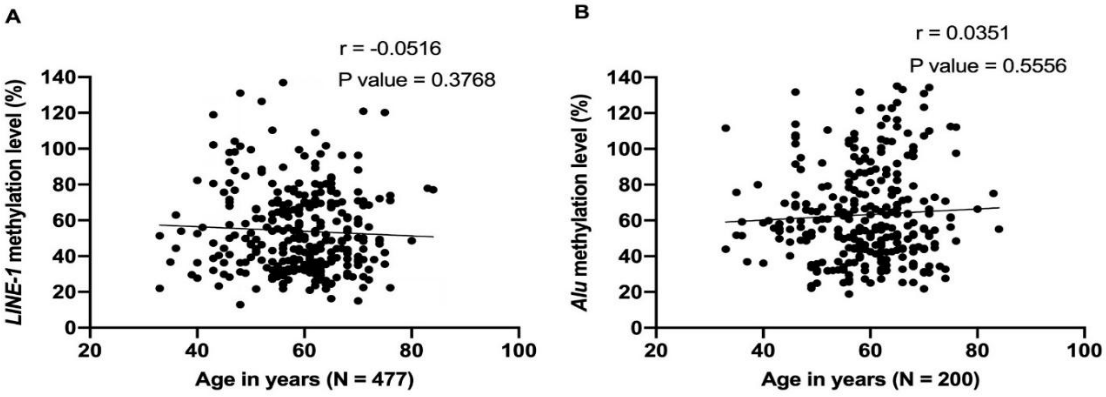

S4 Fig. *LINE-1* (**A**) and *Alu* (**B**) methylation levels in cfDNA do not correlate with ageing in lung cancer patients. Methylation assessments were performed on one microlitre of bisulfite-converted cfDNA. The Spearman's rank correlation test (A, B) was used in statistical analysis.
