# Supplementary material for: An appropriate DNA input for bisulfite conversion reveals LINE-1 and Alu hypermethylation in tissues and circulating cell-free DNA from cancers
Source: PLoS One. 2024 Dec 30;19(12):e0316394. doi: 10.1371/journal.pone.0316394 (PMC11684646; doi:10.1371/journal.pone.0316394)
Supplement: S5 Fig — No relationship was observed between the copy numbers of short LINE-1 (A) and short Alu (B) with their methylation level in both cfNC and cfLC. A negative correlation was detected between the long Alu copy number and Alu methylation in cfNC but not in cfLC (C). Alu Index negatively correlated with Alu methylation in both cfNC and cfLC (D). Correlations were assessed using Spearman’s rank correlation test. (PDF) [file pone.0316394.s008.pdf]

S5 Fig: An appropriate DNA input for bisulfite conversion reveals *LINE-1* and *Alu* hypermethylation in tissues and circulating cell-free DNA from cancers

Trang Thi Quynh Tran[1,2], Tung The Pham[1], Than Thi Nguyen[1,4], Trang Hien Do[1], Phuong Thi Thu Luu[1], Uyen Quynh Nguyen[2], Linh Dieu Vuong[3], Quang Ngoc Nguyen[3], Son Van Ho[4], Hang Viet Dao[5], Tong Van Hoang[6], Lan Thi Thuong Vo[1,2*]

1 Faculty of Biology, VNU University of Science, Vietnam National University, Hanoi. 2 VNU Institute of Microbiology and Biotechnology. 3 Pathology and Molecular Biology Center, Vietnam National Cancer Hospital. 4 Department of Chemistry, 175 Hospital, Ho Chi Minh City. 5 Endoscopic Centre, Hanoi Medical University Hospital. 6 Institute of Biomedicine and Pharmacy, Ha Dong, Vietnam.

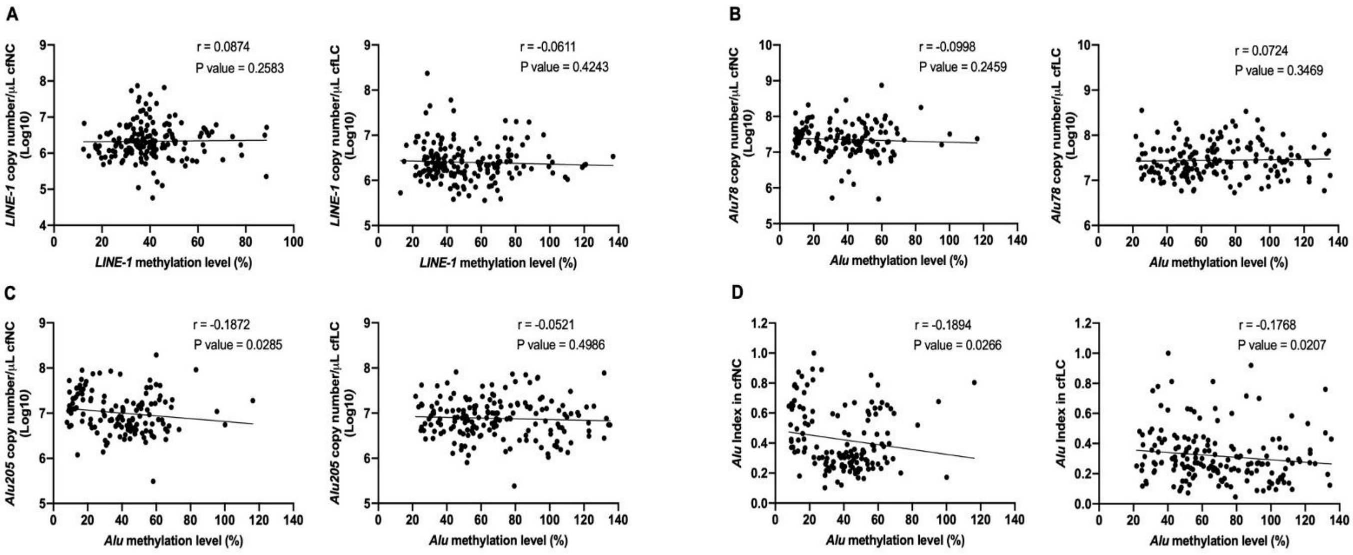

S5 Fig. Correlation between *LINE-1* and *Alu* fragmentation sizes with their methylation level in cfDNA from healthy individuals (cfNC) and lung cancer patients (cfLC). No relationship was observed between the copy numbers of short *LINE*-1 (A) and short *Alu* (B) with their methylation level in both cfNC and cfLC. A negative correlation was detected between the long *Alu* copy number and *Alu* methylation in cfNC but not in cfLC (C). *Alu* Index negatively correlated with *Alu* methylation in both cfNC and cfLC (D). Correlations were assessed using Spearman's rank correlation test.
