# Supplementary material for: An appropriate DNA input for bisulfite conversion reveals LINE-1 and Alu hypermethylation in tissues and circulating cell-free DNA from cancers
Source: PLoS One. 2024 Dec 30;19(12):e0316394. doi: 10.1371/journal.pone.0316394 (PMC11684646; doi:10.1371/journal.pone.0316394)
Supplement: Attachment — Submitted filename: Response to PLoS ONE Editorial Board-Tran et al.docx [file pone.0316394.s010.docx]

Vo Thi Thuong Lan, Assoc. Prof.

Faculty of Biology, VNU University of Science,

334, Nguyen Trai, Thanh Xuan

Ha Noi, Viet Nam.

**PLoS ONE Editorial Board**

Ha Noi, November 27^th^, 2024

Dear Members of the Editorial Board,

We sincerely appreciate the guidance from the Editors and the comments from the Reviewers throughout the review process to improve our manuscript titled “**An appropriate DNA input for bisulfite conversion reveals *LINE-1* and *Alu* hypermethylation in tissues and circulating cell-free DNA from cancers**” by Tran *et al*., submitted to **PLoS ONE** under the reference ID: PONE-D-24-37696R1.

As per the journal requirements kindly provided by the Editor, we have now revised the manuscript to address these points. Modifications have been tracked in the revised manuscript and are detailed point by point in the following section.

To better adhere to the journal requirements, we have:

- Ran our figures through the PACE tool for renaming and reformatting to meet technical requirements
- Confirmed NO retracted paper was cited in our manuscript.
- Outlined the specifics of the corrections for the five papers that have undergone author correction cited in our manuscript and the final state of these references in the revised manuscript, presented in the table below.

We are truly grateful for your time and efforts.

Best regards,

| **Reference with Author Correction** | **Published Author Correction** | **Correction** | **Final citation in manuscript** |
| --- | --- | --- | --- |
| Ref. 15 | *Nature Genetics.* Published online February 5^th^, 2020. doi: [10.1038/s41588-019-0562-0](https://doi.org/10.1038/s41588-019-0562-0) | In the published version of this paper, the members of the Pan-Cancer Analysis of Whole Genomes (PCAWG) Consortium were listed in the Supplementary Information. In the corrected version, these members have been included in the main paper. The original Article has been corrected to include the members and affiliations of the PCAWG Consortium in the main paper. | Ref. 15 |
| Ref. 18 | *Nature.* 2019 Aug; 572(7767): E5. doi: [10.1038/s41586-019-1350-9](https://doi.org/10.1038/s41586-019-1350-9) | In this article, owing to a conceptual error, the standard deviations have been recalculated and all affected figures have been corrected online and the corresponding Source Data files have been updated. These corrections do not affect any of the experimental results, their interpretation or the conclusions of the paper. | Ref. 18 |
| Ref. 23 | *Bioinformatics.* 2024 Oct;18;40(10):btae600. doi: [10.1093/bioinformatics/btae600](https://doi.org/10.1093/bioinformatics/btae600) | In the originally published version of this manuscript, [Supplementary Table S1](https://pmc.ncbi.nlm.nih.gov/articles/PMC11488974/#sup1), [Supplementary Table S2](https://pmc.ncbi.nlm.nih.gov/articles/PMC11488974/#sup1) and [Supplementary Table S4](https://pmc.ncbi.nlm.nih.gov/articles/PMC11488974/#sup1) were omitted in error. These Supplementary Tables do not alter experimental results. | Ref. 23 was cited along with other references (line 70 page 3 and line 552 page 22); thus, we REMOVED its citation. |
| Ref. 45 | [*Genome Res.* 2011 Jan;21(1):146](https://pmc.ncbi.nlm.nih.gov/articles/PMC3012922/). | Panels A and B in Figure 7 were incorrectly reversed in the text and the figure legend. This correction does not affect any of the experimental results. | Ref. 44 (moved up due to removal of Ref. 23) |
| Ref. 53 | *Nucleic Acids Res.* 2009 Aug 4;37(15):5235. doi: [10.1093/nar/gkp637](https://doi.org/10.1093/nar/gkp637) | The correct final bisulfite exposure of DNA in the LowMT protocol was 3.4 M, not 5.5 M. This correction does not affect the citation in our manuscript. | Ref. 52 (moved up due to removal of Ref. 23) |
